# Effectiveness and Safety of Oxcarbazepine vs. Levetiracetam as Monotherapy for Infantile Focal Epilepsy: A Longitudinal Cohort Study

**DOI:** 10.3389/fneur.2022.909191

**Published:** 2022-06-01

**Authors:** Binyang Zhao, Shuang Liao, Xuefei Zhong, Yuanyuan Luo, Siqi Hong, Min Cheng, Jie Zhang, Tingsong Li, Li Jiang

**Affiliations:** ^1^Chongqing Key Laboratory of Pediatrics, Ministry of Education Key Laboratory of Child Development and Disorders, Department of Neurology, National Clinical Research Center for Child Health and Disorders, International Science and Technology Cooperation Base of Child Development and Critical Disorders, Children's Hospital of Chongqing Medical University (CHCMU), Chongqing, China; ^2^Department of Rehabilitation, Children's Hospital of Chongqing Medical University, Chongqing, China; ^3^Editorial Department of Journal of Pediatric Pharmacy, Children's Hospital of Chongqing Medical University, Chongqing, China; ^4^Liangping Maternal and Child Health Hospital, Chongqing, China

**Keywords:** focal epilepsy, infant, oxcarbazepine, levetiracetam, cohort study

## Abstract

**Objective:**

This study aimed to compare the effectiveness and safety of oxcarbazepine (OXC) vs. levetiracetam (LEV) for treating infantile focal epilepsy in a longitudinal cohort study.

**Methods:**

We enrolled 187 consecutive patients aged 2–24 months who received OXC or LEV as initial monotherapy; 161 patients completed the study. The longitudinal analysis involved anti-seizure medication (ASM) responsiveness, safety, the establishment of epilepsy syndrome, and etiology over a median follow-up of 2 years (interquartile range [IQR] 1.6–2.4). The relative efficacy and retention rates of OXC vs. LEV were evaluated using generalized linear regression models and the Cox proportional hazards model.

**Results:**

The 161 patients who completed the study had comparable baseline demographics and clinical variables between the OXC group (*n* = 83) and LEV group (*n* = 78). Overall, the mean age at onset was 6 months (IQR 4.3–9). The most common epilepsy syndrome was self-limited familial/non-familial infantile epilepsy (54.7%). Epilepsy was related to genetic and unknown causes in 34.2 and 52.2% of the patients, respectively. OXC achieved significantly higher responses than LEV for seizure freedom (risk ratio [RR] = 1.71, 95% confidence interval [CI] = 1.28–2.73, *P* < 0.001) and 12-month retention rate after onset (hazard ratio [HR] = 1.84, 95% CI = 1.15–2.95, *P* = 0.007). Moreover, OXC showed more obvious effects for patients aged < 1 year diagnosed with self-limited familial/non-familial infantile epilepsy and non-syndromic epilepsy with genetic or unknown causes. The adverse events related to both OXC and LEV were well-tolerated.

**Significance:**

OXC could be an alternative to LEV for treating infantile focal epilepsy. OXC monotherapy can be considered first-line treatment for patients aged <12 months and those with epilepsy without developmental and epileptic encephalopathy.

## Introduction

The incidence of epilepsy in the infantile period is highest among all age groups and affects an estimated 70.1 per 100,000 children ≤2 years of age (1). Up to 72% of childhood epilepsy is a partial seizure, namely focal-onset seizures, whether secondarily generalized or not (2). Oxcarbazepine (OXC) has been established as the first-line medication choice (Level A) for children with partial-onset seizures (3). By comparison, levetiracetam (LEV) is recommended for infantile focal seizures, with strong efficacy, while OXC has been deemed ineffective (1) based on the previously reported findings (4–6). Notably, a retrospective study reported that 73% (8/11) of infantile focal epilepsy on initial OXC monotherapy achieved complete seizure freedom (6). However, there are limited data on the efficacy and tolerability of OXC as monotherapy for infantile focal epilepsy, nor has there been a comparative study between OXC and LEV in this respect.

Although it has been acknowledged that the results of efficacy trials performed in adults can be extrapolated to children aged >4 years (7), this approach is not well-applied for childhood epilepsy because of the neglect of epilepsy syndrome (8). The etiology of focal epilepsy in infancy is largely heterogeneous, consisting of all the causes proposed by the International League Against Epilepsy (ILAE) in 2017 (9). Moreover, it is advisable to avoid introducing OXC for some genetic epilepsy, particularly in *SCN1A*-related Dravet syndrome, in spite of its prominent characteristics of focal seizures. Therefore, full consideration should be given to the exclusive epilepsy syndromes and underlying etiologies during childhood when assessing the efficacy, safety and tolerability, and pharmacokinetics of anti-seizure medication (ASM) in children.

In the present study, we evaluated the efficacy and safety of OXC as monotherapy by comparing it with the acknowledged first-line therapy LEV through a longitudinal cohort study. We also explored the potentially relevant factors influencing responsiveness to ASMs.

## Materials and Methods

This was a single-center, ambispective and observational cohort study. It was conducted from February 2015 to April 2019 at the Children's Hospital of Chongqing Medical University (CHCMU). Data analysis was performed from May 1 to July 30, 2020. The study was approved by the CHCMU Institutional Review Board and it has been registered with the Chinese Clinical Trial Registry (ChiCTR1900028463). Informed consent was obtained from the parents or legal guardians of each patient.

### Patients

All consecutively enrolled patients were aged 2–24 months and newly diagnosed with focal epilepsy without any ASMs before admission to the outpatient department and neurology ward. The diagnoses of focal epilepsy were established according to focal-onset ictal events on EEG and/or the cranial MRI with lesions indicative of focal seizures. Otherwise, focal epilepsy can be diagnosed only by an experienced pediatric neurologist due to the observed episodes or related videos which presented repetitive and stereotyped events. The focal onset motor epileptic seizures were recorded based on the instruction manual for the ILAE 2017 operational classification of seizure types (10). The inclusion criteria were the following: (1) patients receiving OXC or LEV as initial monotherapy and (2) were followed up for at least 1 year by pediatric neurologists. The exclusion criteria were the following: (1) patients were contraindicated to OXC or LEV listed in their datasheet; (2) patients with potential insufficient information to assess if a seizure occurred; (3) patients had a risk of being lost during follow-up; (4) patients had previous treatment with other ASMs beyond OXC and LEV; (5) patients receiving OXC presented as febrile convulsion in the beginning to avoid the bias due to the fact that OXC can potentially exacerbate the underlying SCN1A-related epilepsy. The demographic data were reviewed retrospectively *via* the medical records.

### Study Design

The decision to administer OXC or LEV was based on clinical presentation, physicians' judgment, and parents' preferences. Patients were interviewed for the first time 1 month after the introduction of any medications, then regularly followed up at the outpatient clinic or by telephone every 3 months. Besides seizures, neuropsychological conditions, including fine and gross motor, language, adaptive personal/social skills, and other vital signs, and related laboratory findings were also recorded at follow-up. The medication regimen consisted of a starting dose of 10 mg/kg/d of each ASMs, up to a maintenance maximal dose of around 40 mg/kg/d for both OXC and LEV, based on the patient's clinical response and tolerance to treatment. The titration duration was up to 4 weeks. If the seizures were not well-controlled, concomitant or switched treatment was permitted. The matched items between the two groups were: sex, onset age, epilepsy syndrome, and epilepsy etiology. Medication-related data included the time from first seizure to ASM introduction, dose, duration of therapy, adverse effects (AEs), reasons for discontinuation, and combination with other medications. Epilepsy syndromes and etiologies were classified according to the ILAE classification of epilepsies (9, 11).

### Outcome Measures

The primary outcome measure was the percentage of seizure freedom for ≥12 months. The 12-month seizure freedom was defined as patients having no seizures for at least 12 months after achieving the maintenance dose (12). The secondary outcome was retention rate, defined as the proportion of previous ASMs being continued with no change in the therapy regimen (13). The secondary outcome is measured based on the following two aspects: (1) The number of patients maintaining previous therapy at established time points (i.e., 1, 3, 6, 12 months after receiving medication); and (2) The time to discontinuation of ASMs, defined as the duration from ASMs introduction until discontinuation due to AEs, lack of efficacy, or the need for other treatment (added or switched).

The safety and tolerability assessments included the number and percentage of AEs and severe AEs (SAEs). An AE was defined as any untoward or unintended response thought to be related to the treatment. An AE was deemed serious if it was life-threatening, caused death, resulted in persistent or substantial disability, or required admission to hospital (14).

### Statistical Analysis

The sample size calculations were based on the effective rate for comparing OXC with LEV. From the results of previous studies, the effective rate of OXC and LEV was 73% (6) and 46.9% (15), respectively. We set the patient number ratio as 1:1, assuming a 15% dropout rate. Therefore, to statistically satisfy 90% power and 95% confidence, the estimated sample size in each group was 82 (164 in total).

We performed comparative statistics with the chi-square test for categorical variables, and with the Mann-Whitney U tests for skewed-distribution continuous variables. The associations between treatment with seizure outcome were examined using generalized linear regression models consisting of logistic regression, binomial distribution, and the delta method. Risk ratios (RRs) and 95% CIs were estimated to quantify the strength of the association. The basic model was adjusted for sex, and the time from first seizure to ASMs administration. The confounder model was additionally adjusted for onset age, etiology, and syndrome. These confounders were selected based on their associations with the outcome of interest or change in effect estimate of > 10% (16). Supplementary Table 1 shows the associations of each confounder with the outcome of interest. Subgroup analysis was performed to verify the robustness of the results. The results are depicted using a forest plot. The time to discontinuation between the two treatment groups was compared using Kaplan–Meier survival analysis. The influence of the treatments on retention rate was assessed using the Cox proportional hazards model. Sensitivity analyses about patient withdrawal were performed to ensure the robustness of the data analysis (Supplementary Table 2).

Two-sided *P*-values of <0.05 were considered statistically significant. All analyses were conducted using the statistical package R (http://www.r-project.org, R Foundation, Vienna, Austria) and EmpowerStats software (www.empowerstats.com, X&Y Solutions, Inc., Shanghai, China).

## Results

### Baseline Demographics and Epilepsy-Specific Variables

**Figure 1:**
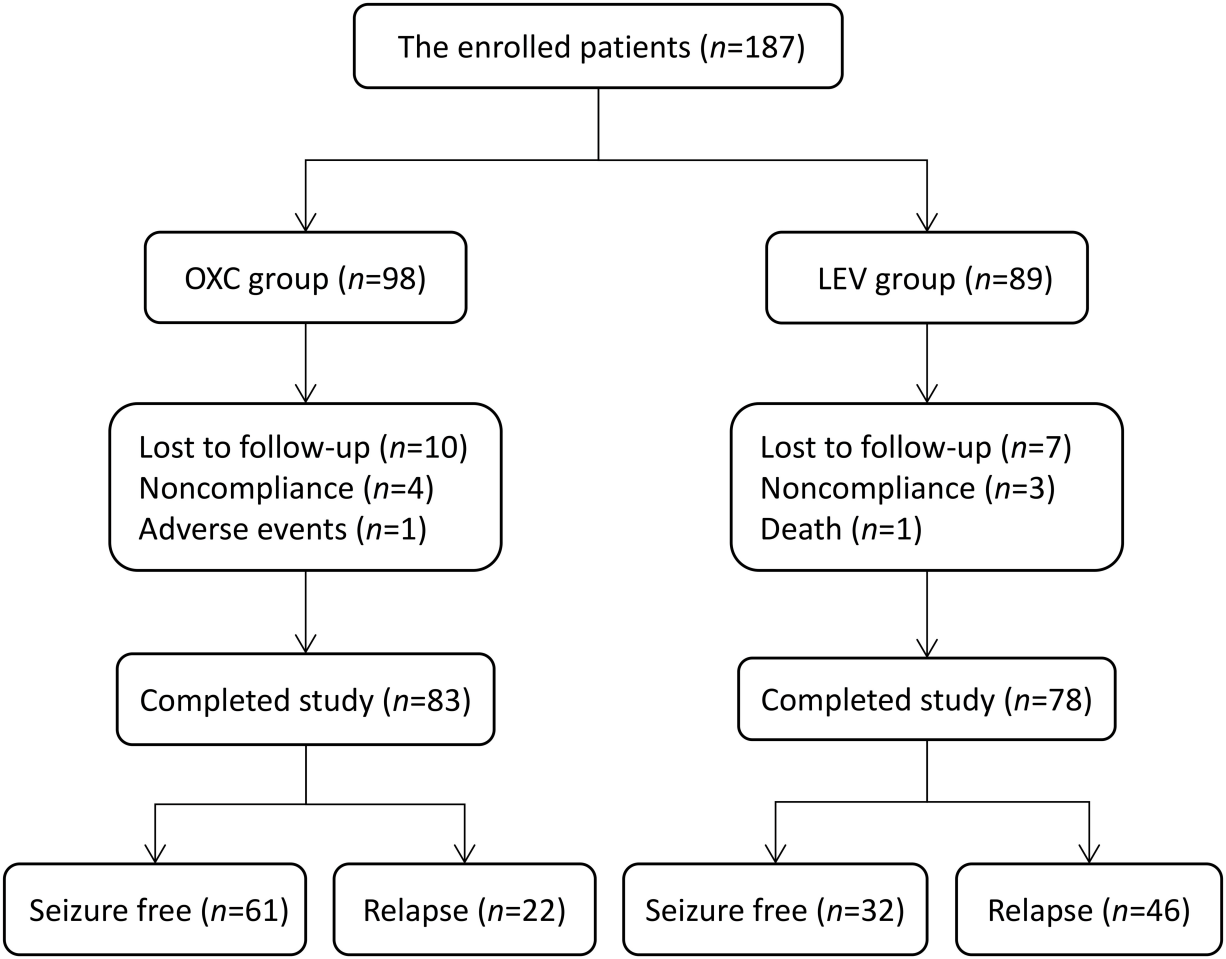
Flowchart depicting the overall outcomes of the study cohort. OXC, oxcarbazepine; LEV, levetiracetam.

We enrolled 356 patients in total aged 2–24 months with focal epilepsy from February 2015 to April 2019. One hundred and eighty seven patients who received OXC or LEV as the first monotherapy underwent evaluations for eligibility. Forty five cases out of them were retrospectively analyzed and the remaining 142 subjects were followed up prospectively. All the five *SCN1A* variants associated with epilepsy were allocated to the LEV group as shown in Table 1. Of these patients, 26 (13.9%) withdrew from the cohort during follow-up, with the following reasons: lost to follow-up (*n* = 17), non-compliance (*n* = 7), SAEs (*n* = 1), and death (*n* = 1). Finally, 161 patients completed the study and were enrolled in the effectiveness assessment (Figure 1).

**Table 1:** Patient baseline demographics and clinical characteristics (*n* = 161).

	**Total**	**OXC**	**LEV**	
**Characteristic**	***n* = 161**	***n* = 83**	***n* = 78**	***P***
Male, *n* (%)	68 (42.2)	38 (45.8)	30 (38.5)	0.35
Age at onset, mo, median (IQR)	6.0 (4.3–9.0)	7.0 (4.8–9.0)	5.4 (4.0–9.0)	0.08
Age at onset, mo, *n* (%)				0.95
2–12 m	138 (85.7)	71 (85.5)	67 (85.9)	
<24 m	23 (14.3)	12 (14.5)	11 (14.1)	
Epilepsy etiology, *n* (%)				0.24
Genetics	55 (34.2)	23 (27.7)	32 (41.0)	
PRRT2	11	4	7	
SCN1A	5	0	5	
KCNQ2	1	1	0	
KCNT1	1	0	1	
TBC1D24	1	1	0	
SLC9A6	1	1	0	
EFTUD2	1	0	1	
Microdeletion	2	1	1	
Unclear	32	15	17	
Structural	15 (9.3)	11 (13.3)	4 (5.1)	
Sturge-Weber syndrome	3	3	0	
Hypoxic-ischemic encephalopathy	3	1	2	
Hippocampal lesions	2	2	0	
Tuberous sclerosis	3	2	1	
Intracranial hemorrhage	2	1	1	
Corpus callosum lesion	2	2	0	
Infectious	5 (3.1)	2 (2.4)	3 (3.8)	
Bacterial meningitis	3	1	2	
Viral meningitis	2	1	1	
Metabolic	2 (1.2)	1 (1.2)	1 (1.3)	
Mitochondrial encephalopathy	1	0	1	
Menkes' disease	1	1	0	
Unknown	84 (52.2)	46 (55.4)	38 (48.7)	
Epilepsy syndrome, *n* (%)				0.24
SLNFIE	60 (37.3)	28 (33.7)	32 (41.0)	
SLFIE	28 (17.4)	15 (18.1)	13 (16.7)	
DEE	23 (14.3)	8 (9.6)	15 (19.2)	
NSE	50 (31.1)	32 (38.6)	18 (23.1)	
Seizure type, *n* (%)				0.31
Only focal seizure	83 (51.6)	46 (55.4)	37 (47.4)	
Focal to bilateral tonic-clonic	78 (48.5)	37 (44.6)	41 (52.6)	
Time since the first seizure to ASM, d, median (IQR)	15.0 (5.0–58.0)	15.0 (4.5–32.5)	17.0 (6.3–60.0)	0.78
Maintenance dose, mg/kg/d, median (IQR)	–	30.0 (25.0–35.5)	30.0 (25.0–40.0)	
Follow-up, yrs, median (IQR)	2.0 (1.6–2.4)	2.0 (1.8–2.4)	1.6 (1.4–2.4)	0.02

*OXC, oxcarbazepine; LEV, levetiracetam; SLNFIE, non-familial infantile epilepsy; SLFIE, self-limited familial infantile epilepsy; DEE, developmental and epileptic encephalopathies; NSE, non-syndromic epilepsy; IQR, interquartile range*.

Table 1 shows the demographics and epilepsy-related variables at baseline in the 161 patients. A total of 83 and 78 patients were treated with OXC and LEV, respectively, as the initial monotherapy. There was no significant difference between the two groups for sex, onset age, time from first seizure to ASMs introduction, etiology, and epilepsy syndrome. Overall, the mean patient age was 6 months (interquartile range [IQR] 4.3–9), and 68 patients (42.2%) were men. Regarding etiological analysis, 34.2% of the patients (55/161) had a family history of epilepsy, among which 14.3% (23/161) had pathogenic genetic variants, 9.3% (15/161) had structural brain abnormalities, 3.1% (5/161) were categorized as being caused by previous central nervous system infection, 1.2% (2/161) had the metabolic disease, and 52.2% (84/161) was due to unknown cause. On the distribution of epilepsy syndromes, 37.3% of the cases (60/161) had self-limited non-familial infantile epilepsy (SLNFIE), 17.4% (28/161) had self-limited familial infantile epilepsy (SLFIE), 14.3% (23/161) had developmental and epileptic encephalopathies (DEE), and 31.1% (50/161) had non-syndromic epilepsy (NSE). Self-limited infantile epilepsy (SLIE), which includes both SLNFIE and SLFIE, was the most commonly diagnosed epilepsy syndrome (*n* = 88, 54.7%). The median follow-up period was 2 years (IQR 1.6–2.4).

### Effectiveness

#### Seizure Freedom Rates With Successive Monotherapy

The 12-month seizure freedom rate since the start of OXC and LEV therapy was 73.5% (61/83) and 41% (32/78), respectively. Table 2 shows the generalized linear regression analysis of the association between the treatments and seizure outcome. The basic model showed that OXC-treated patients had a 1.79-fold (95% CI = 1.35–2.5, *P* < 0.001) increased probability of seizure freedom than LEV-treated patients. The confounder model presented 1.71-fold (95% CI = 1.28–2.73, *P* < 0.001) increased probability. After adjusting for onset age, etiology, and syndrome, the associations did not change markedly. The results suggest that OXC is more effective for seizure control than LEV.

**Table 2:** Generalized linear regression models evaluating the association between treatment and seizure outcome (*n* = 161).

	**Total**	**Seizure-free**	**Basic model**		**Confounder model**	
	***n***	***n* (%)**	**Risk ratio**	***P***	**Risk ratio**	***P***
Levetiracetam	78	32 (41.0)	Ref	Ref	Ref	Ref
Oxcarbazepine	83	61 (73.5)	1.79 (1.35–2.50)	<0.001	1.71 (1.28–2.73)	<0.001

*Values are regression coefficients (95% confidence interval) that reflect the difference in seizure outcomes with treatment. The basic model was adjusted for sex and the time from first seizure to ASM. The confounder model was additionally adjusted for onset age, etiology, and syndrome*.

#### Factors Associated With Efficacy

**Figure 2:**
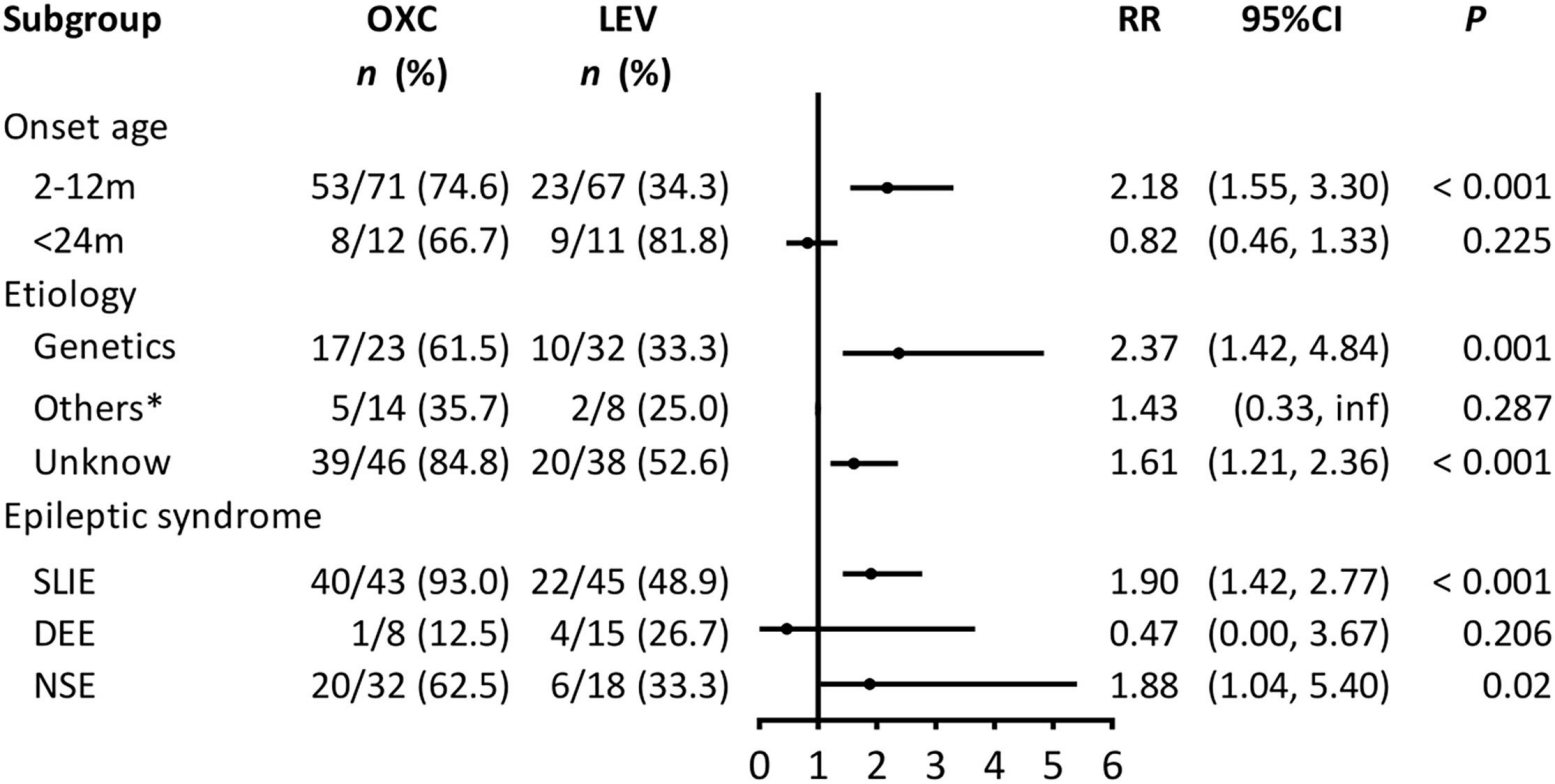
Subgroup analysis of the association between treatment and seizure outcome (*n* = 161). OXC, oxcarbazepine; LEV, levetiracetam; CI, confidence interval; RR, risk ratio; SLIE, self-limited infantile epilepsy; DEE, developmental and epileptic encephalopathies; NSE, non-syndromic epilepsy. Others*: includes structural, infectious, metabolic causes; we could not calculate the 95% CI because of the small sample size.

Subgroup analyses were conducted to explore the factors associated with the efficacy of each regimen. Due to the small sample size, the structural, infectious, and metabolic etiology subgroups were combined into the “others” subgroup. Figure 2 shows the association between the treatments and seizure outcome in the stratified groups. OXC was more effective for achieving seizure freedom in the subgroups for patients aged 2–12 months (RR = 2.18, 95% CI = 1.55–3.3, *P* < 0.001), genetics (RR = 2.37, 95% CI = 1.42–4.84, *P* = 0.001), unknown etiology (RR = 1.61, 95% CI = 1.21–2.36, *P* < 0.001), SLIE (RR = 1.9, 95% CI = 1.42–2.77, *P* < 0.001), and NSE (RR = 1.88, 95% CI = 1.04–5.4, *P* = 0.02).

#### Overall Seizure Outcome According to the Treatment Regimen

Among the 22 patients with seizure relapse in the OXC group, eight with irregular titration due to poor compliance (36.4%) continued with OXC, with no subsequent relapse in six patients. Of the remaining two patients, one (4.5%) switched to LEV and one (4.5%) underwent surgery; both achieved seizure freedom. A total of 12 patients (54.5%) chose to combine other ASMs, whereas only two were seizure-free. Until the end of our study, the eventual seizure-free rate in the OXC group was 85.5% (71/83).

Likewise, of the 46 patients with seizure relapse in the LEV group, 14 (30.4%) continued regular oral LEV as advised by their doctor, of which 10 had no subsequent relapse; five patients (10.9%) switched to OXC, and all achieved seizure freedom; another five (10.9%) switched to valproate (VPA), of which four achieved seizure freedom, while one patient (2.2%) underwent surgery and achieved seizure freedom. Twenty-one patients (45.7%) chose to combine ASMs, of which nine achieved seizure freedom. At the last follow-up, the eventual seizure-free rate in the LEV group was 78.2% (61/78). There was no difference in the overall seizure-free rate between the two groups (χ^2^ = 2.04, *P* = 0.15), which suggests that the two groups share similar characteristics and consequent medication responses (Supplementary Table 3).

### Retention Rates

**Figure 3:**
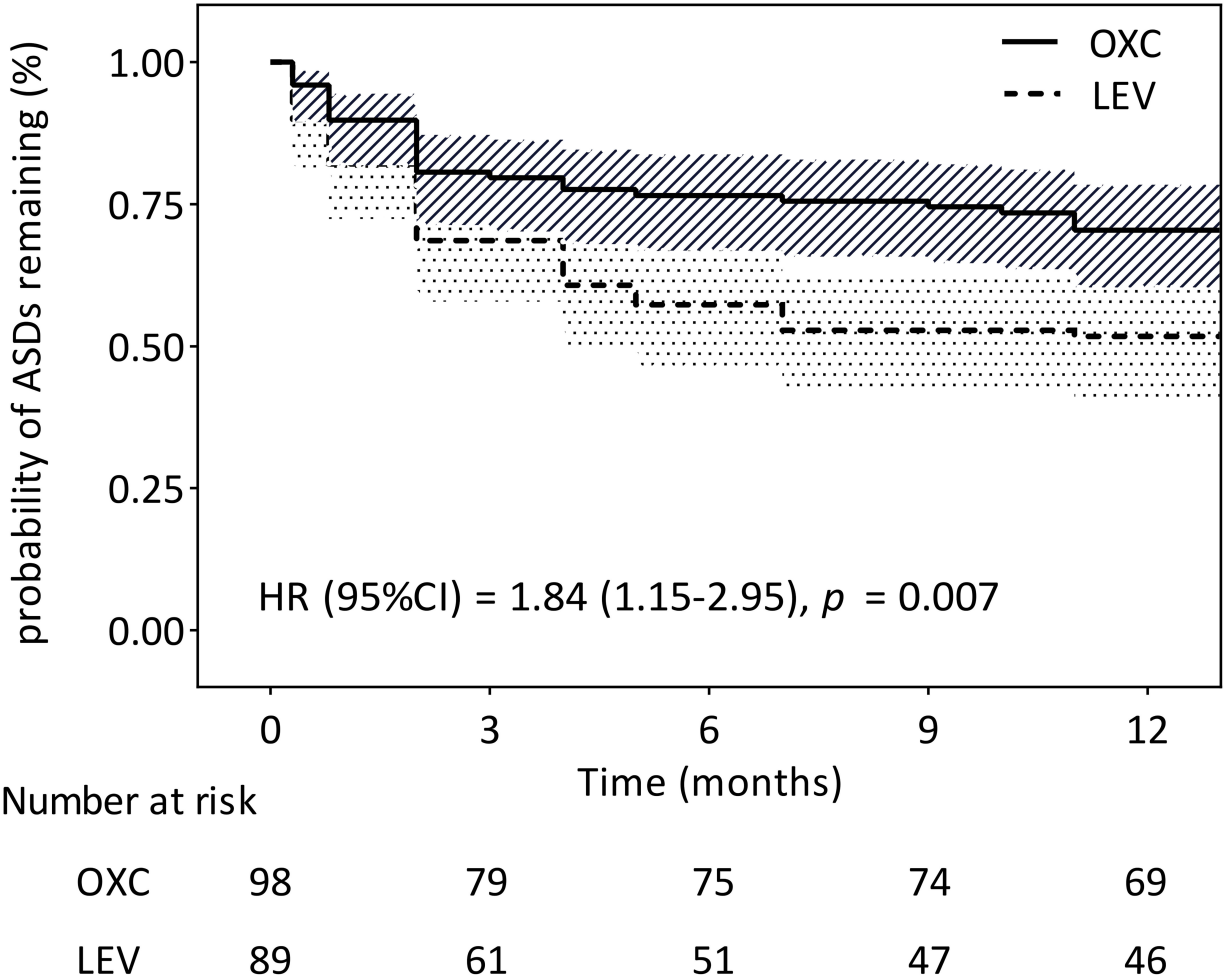
Kaplan–Meier survival analysis of discontinuation between the two treatment groups (*n* = 187). CI, confidence interval; HR, hazard ratio.

Among the 187 patients enrolled in the study, 115 (61.5%) maintained the initially prescribed ASMs without alternation. The 12-month retention rates for OXC and LEV were 70.4% (69/98) and 51.7% (46/89), respectively (Figure 3). The Cox proportional hazard ratio (HR) was 1.84 (95% CI = 1.15–2.95, *P* = 0.007), suggesting that the OXC group had a significantly higher probability of retention than the LEV group. The reasons for discontinuing OXC compared with LEV were the following: lack of efficacy (14.3 vs. 36%), lost to follow-up (10.2 vs. 7.9%), AE (1 vs. 0%), non-compliance (4.1 vs. 3.4%), and death (0 vs. 1.1%).

### Safety and Tolerability

Table 3 shows that AEs were reported in 11 patients (5.9%): eight from the OXC group and three from the LEV group. The most common AEs for LEV were nervous system upset (100%), including somnolence or irritation. Rash (*n* = 3) and medication-induced hypersensitivity syndrome (*n* = 1) accounted for 50% of AE cases (4/8) in the OXC group. All AEs were mild and transient, and responded to symptomatic treatment, except for the patient who experienced medication-induced hypersensitivity syndrome, leading to the discontinuation of OXC. One patient in the LEV group died, which was not related to the medication. Laboratory tests, such as liver and kidney function, electrolytes, and blood routine testing, did not demonstrate clinically significant changes.

**Table 3:** Reported adverse events (AEs) (*n* = 187).

		**OXC**	**LEV**
		***n* (%)**	***n* (%)**
AEs	Rash	3 (3.1)	0
	DIHS	1 (1.0)	0
	Somnolence	2 (2.0)	1 (1.1)
	Excitement	0	1 (1.1)
	Irritation	1 (1.0)	1 (1.1)
	Vomit	1 (1.0)	0

*AEs, adverse events; DIHS, drug-induced hypersensitivity syndrome*.

## Discussion

The present longitudinal cohort study shows that OXC as monotherapy for focal-onset epilepsy in infancy was significantly more effective for achieving seizure freedom and a 12-month retention rate than LEV, especially for patients aged < 1 year and diagnosed with SLIE or NSE. Furthermore, OXC was generally well-tolerated in infants, with most AEs rated as mild in severity and only one patient discontinuing OXC due to medication-induced hypersensitivity syndrome. To our knowledge, this is the first study comparing the effectiveness of OXC and LEV in infantile epilepsy, the most two common ASMs for focal seizures, which suggests that OXC could be an alternative to LEV for treating infantile focal epilepsy, due to the apparent higher efficacy and well-tolerated adverse reactions.

Epilepsy syndromes have age-dependent features and associated etiological, prognostic, and treatment implications. In the present study, the matched items between the two groups, i.e., epilepsy syndrome, etiology, and onset age, were set as comparable to minimize selective bias. We did not include West syndrome because its first recommended medication is ACTH (adrenocorticotrophic hormone) or glucosteroids, rather than ASMs (1). We also did not enroll *SCN1A*-related epilepsy cases in the OXC group, considering the exacerbation of epileptic seizures due to the loss of function of Na_v_1.1 (sodium voltage-gated channel alpha subunit 1) in most cases with pathogenic variants (17). Moreover, both treatment groups had similar duration from seizure onset to ASM introduction and overall response to ASMs. Therefore, the two cohorts in our study may be considered as deriving from the same population without contradiction to OXC or LEV administration.

A US database analysis showed that only 3.5% of children aged < 1 year and 14.8% of chidden aged 1–4 years were prescribed OXC, which was much lower than the rate of children prescribed LEV, being 32.7 and 58.1%, respectively (18). A randomized controlled trial (RCT) in 2009 by Piña-Garza et al. demonstrated that the responder rate was 43.1% for LEV as adjunctive therapy in infants and young children with refractory partial-onset seizures, which was significantly higher than the 19.6% for placebo (19). In 2015, the ILAE graded LEV as a strong recommendation for infantile focal epilepsy, while assessing OXC as ineffective (1). However, a rater-blind, randomized, parallel-group adjunctive therapy study showed that the median percentage reduction was greater in the high-dose OXC group (60 mg/kg/d) than in the low-dose group (10 mg/kg/d) (83.33 vs. 46.18%) (4). The results confirm that OXC is a valuable treatment option for managing infants and young children (<4 years old) with partial seizures. Likewise, we show that the efficacies of LEV and OXC as monotherapy for focal-onset infantile epilepsy were 41 and 73.5%, respectively, which is similar to previous reports (6, 19). Importantly, our data show that OXC efficacy, defined as seizure freedom > 1 year, and 12-month retention rate were significantly higher than that of LEV, which not only confirms that OXC is a valuable treatment option but also provides evidence for the beneficial choice of OXC as monotherapy for infants with focal-onset epilepsy.

The subgroup analysis, aimed at exploring the factors related to the different outcomes, revealed that OXC was more effective than LEV for SLIE. Kikuchi et al. foundthat of 23 SLIE patients (78.3%) treated with ASMs, 18 remained seizure-free after discontinuing the ASMs (20). In the present study, the efficacy of OXC for SLIE was 93%, higher than that reported by Kikuchi et al. (20), in which the most frequent ASM was VPA, rather than OXC or LEV. The higher efficacy of OXC than LEV for SLIE may be explained by the fact that *PRRT2* variant-associated SLIE, which accounts for > 90% of SLIE cases (21), has a better response to OXC than LEV (22, 23). We also found that, from the aspect of etiology, OXC was more effective than LEV for cases with genetic and unknown causes. One study identified pathogenic or likely pathogenic variants for 40% of pediatric epilepsy with unknown causes at the onset (24). Moreover, the most prevalent genetic diagnoses for infantile epilepsy, particularly for patients aged < 12 months, were *KCNQ2, PRRT2*, and *SCN1A* (25). Importantly, sodium channel blockers, such as OXC and carbamazepine, have proven to be efficacious in *PRRT2*- and *KCNQ2*-related infantile epilepsies (25). By comparison, the response to LEV was inferior in *PRRT2*- and *KCNQ2*-related epilepsy, and it may probably be useful for *SCN1A*-associated epilepsy (26). In the present study, we did not include cases with *SCN1A*-related epileptic encephalopathies in the OXC group, so that the iatrogenic exacerbation of seizures and the subsequent outcome bias could be avoided. Therefore, the greater advantageous effects of OXC over LEV for infantile epilepsy in patients aged < 1 year with genetic or unknown causes and non-DEE epileptic syndromes may be ascribed to the distribution of genetic etiology in younger children and the presumptive precise ASMs choice. According to the recommended ASM monotherapy for initial treatment by ILAE (1), OXC or LEV monotherapy as a start intervention was given in this cohort. Of note, 23 patients were diagnosed with DEE during observation which tends to evolve into pharmacoresistant epilepsy even from the onset. Actually, the initial ASMs monotherapy for all the 23 patients were eventually switched to other ASMs or adjusted to ASMs combination. Therefore, the cases with diagnosed or suspected DEE should be evaluated comprehensively during illness courses and thereby optimized therapeutic regiments accordingly.

For LEV, only three cases (3.3%) presented with somnolence, or irritation. The lower percentage of adverse reactions than that reported in another study (19) may be due to the lower dose in our study. It has been reported that there were apparent dose-dependent LEV-induced psychiatric disorders in some cases (27) and increased neuronal apoptosis in the neonatal mouse brain (28). We, therefore, set the highest dose of LEV as around 40 mg/Kg/d in this study aiming to avoid the potential side effects as far as possible. In line with a study that enrolled children aged 2–5 years (29), the most common AEs by OXC were rash (3.1%) and somnolence (2%). All AEs were of mild severity and were transient; the exception was one patient who withdrew from the OXC group due to medication-induced hypersensitivity syndrome. All data indicate that both OXC and LEV are well-tolerated within the therapeutic dosage and that younger age is not a risk factor for AEs.

There are some limitations to this study. Firstly, the enrolled patients were from one single tertiary pediatric epilepsy center, which could limit the extension of the findings. Secondly, more than half of the patients were diagnosed with SLNFIE and SLFIE in this cohort study, which may have exaggerated the responses of OXC and LEV because of the self-limited characteristics of the two syndromes. Thirdly, we might also have underestimated the AEs, as some patients were followed by telephone, and young children are unable to describe subjective symptoms. Lastly, due to the small sample size, the effects of epileptic syndromes and etiologies were not fully evaluated for the efficacy discrepancy of OXC and LEV. However, the two groups were balanced in terms of demographics consisting of the aforementioned factors.

In summary, OXC is more efficacious and well-tolerated for focal-onset epilepsy in early childhood when compared with LEV. Moreover, OXC could be considered the first-line ASMs, particularly for children aged < 1 year diagnosed with focal epilepsy without DEE, with genetic or unknown causes. Although our study provides evidence of the comparative effectiveness of OXC and LEV, there remains a need for RCTs with larger sample sizes, especially with respect to specific epilepsy syndromes and etiologies, due to the limitation of evidence-based data for supporting guideline statements.

## Data Availability Statement

The raw data supporting the conclusions of this article will be made available by the authors, without undue reservation.

## Ethics Statement

The studies involving human participants were reviewed and approved by the CHCMU Institutional Review Board. Written informed consent to participate in this study was provided by the participants' legal guardian/next of kin.

## Author Contributions

TL and LJ contributed to conception and design of the study. LY organized the database. SL performed the statistical analysis. BZ and TL wrote the first draft of the manuscript. XZ, SH, and JZ wrote sections of the manuscript. All authors contributed to manuscript revision, read, and approved the submitted version.

## Funding

This work was supported by the Clinical Research Program-CEpiDB (lcjy2015-10) from CHCMU.

## Conflict of Interest

The authors declare that the research was conducted in the absence of any commercial or financial relationships that could be construed as a potential conflict of interest.

## Publisher's Note

All claims expressed in this article are solely those of the authors and do not necessarily represent those of their affiliated organizations, or those of the publisher, the editors and the reviewers. Any product that may be evaluated in this article, or claim that may be made by its manufacturer, is not guaranteed or endorsed by the publisher.
