# Supplementary material for: Effectiveness and Safety of Oxcarbazepine vs. Levetiracetam as Monotherapy for Infantile Focal Epilepsy: A Longitudinal Cohort Study
Source: Front Neurol. 2022 Jun 1;13:909191. doi: 10.3389/fneur.2022.909191 (PMC9198356; doi:10.3389/fneur.2022.909191)
Supplement: Supplementary file 1 [file Data_Sheet_1.docx]

**Table S1.** Associations of covariates with seizure outcome (*n* = 161).

|  | **Seizure outcome** | | |
| --- | --- | --- | --- |
|  | **Risk ratio** | **95%CI** | ***P*** |
| Sex |  |  |  |
| Male | ref | ref | ref |
| Female | 1.21 | 0.90-1.63 | 0.08 |
| Age |  |  |  |
| 2-12m | ref | ref | ref |
| <24m | 1.34 | 0.98-1.77 | 0.04 |
| Etiology |  |  |  |
| Genetics | ref | ref | ref |
| Others* | 1.43 | 1.07-2.06 | 0.01 |
| Unknow | 0.65 | 0.27-1.14 | 0.08 |
| Syndrom |  |  |  |
| SLIE | ref | ref | ref |
| DEE | 0.31 | 0.08-0.55 | < 0.01 |
| NSE | 0.74 | 0.53-0.98 | 0.02 |
| Time from first seizure to AEDs, days | 1.00 | 0.99-1.00 | 0.36 |
| values are regression coefficients (95% Confidence Interval) from univariate regression models and reflect differences in seizure outcome per unit change of each covariate and for different categories of each covariate as compared to the reference group.  Others*: including structural, infectious, metabolic.  SLIE, self-limited infantile epilepsy; DEE, developmental and epileptic encephalopathies; NSE, non-syndromic epilepsy; CI, confidence interval. | | | |

**Table S2.** Sensitivity comparative analysis between Patients with *versus* without know outcome data (*n* = 187).

| **Characteristic** | **Patients with known outcomes** | **Patients with missing outcome data** | ***P*** |
| --- | --- | --- | --- |
| Number | 161 | 26 |  |
| Male, *n* (%) | 68 (42.2) | 15 (57.7) | 0.14 |
| Age of onset, *n* (%)  2-12m  <24m | 138 (85.7)  23 (14.3) | 21 (80.8)  5 (19.2) | 0.51 |
| Epilepsy etiology, *n* (%)  Genetics  Infectious  Structural  Metabolic  Unknown | 55 (34.2)  5 (3.1)  15 (9.3)  2 (1.2)  84 (52.2) | 6 (23.1)  1 (0.04)  6 (23.1)  3 (11.5)  10 (38.5) | 0.01 |
| Seizure type, *n* (%)  Only focal seizure  Focal to bilateral tonic–clonic | 83 (51.6)  78 (48.5) | 12 (46.2)  14 (53.9) | 0.61 |
| Time since the first seizure to AED, d, median (IRQ) | 15.0 (5.0-58.0) | 6.0 (3.0-30.0) | 0.13 |
| IQR, interquartile range. | | |  |

**Table S3****.** Seizure-free rates with different treatments (*n* =161).

|  | **OXC (*n* = 83)** | | **LEV (*n* = 78)** | |
| --- | --- | --- | --- | --- |
|  |  | ***n* (%)** |  | ***n* (%)** |
| **First choice** |  |  |  |  |
| Initial first monotherapy | OXC | 61/83 (73.5) | LEV | 32/78 (41.0) |
| **Second choice after relapse** | |  |  |  |
| Continue first monotherapy | OXC | 6/8 (75.0) | LEV | 10/14 (71.4) |
| Switch to second monotherapy | LEV | 1/1 (100) | OXC  VPA | 5/5 (100)  4/5 (80.0) |
| Combination | OXC+VPA  OXC+NZP  OXC+LEV+NZP  OXC+LEV+VPA+TPM | 2/7 (28.6)  0/2 (0)  0/2 (0)  0/1 (0) | LEV+OXC  LEV+VPA  LEV+NZP  LEV+TPM  LEV+VPA+NZP  LEV+VPA+TPM  LEV+VPA+TPM+OXC  VPA+OXC  VPA+OXC+TPM  VPA+OXC+LAM | 4/4 (100)  3/5 (60)  1/3 (33.3)  1/2 (50.0)  0/1 (0)  0/1 (0)  0/1 (0)  0/2 (0)  0/1 (0)  0/1 (0) |
| Surgery |  | 1/1 (100) |  | 1/1 (100) |
| The number of eventually seizure-free | | 71/83 (85.5) |  | 61/78 (78.2) |
| *P* for eventually seizure-free 0.15 | | | | |
| OXC, oxcarbazepine; LEV, levetiracetam; VPA, valproate; NZP, nitrazepam; TPM, topamax; LAM, lamotrigine. | | | | |
